# Ohio’s Tobacco Law

**Published:** 1896-06

**Authors:** 


### Ohio's Tobacco Law.

The measure recenly introduced into the Ohio Legislature to prohibit the use and to provide a remedy for the sale of tobacco in any form to minors was passed by unanimous vote of that body and is now a law.

